# Space charge governs the kinetics of metal exsolution

**DOI:** 10.1038/s41563-023-01743-6

**Published:** 2024-01-02

**Authors:** Moritz L. Weber, Břetislav Šmíd, Uwe Breuer, Marc-André Rose, Norbert H. Menzler, Regina Dittmann, Rainer Waser, Olivier Guillon, Felix Gunkel, Christian Lenser

**Affiliations:** 1 Peter Gruenberg Institute – Electronic Materials (PGI-7), Forschungszentrum Juelich GmbH, Juelich, Germany; 2 Institute of Energy and Climate Research – Materials Synthesis and Processing (IEK-1), Forschungszentrum Juelich GmbH, Juelich, Germany; 3 Juelich-Aachen Research Alliance (JARA-FIT), Juelich, Germany; 4 Institute of Mineral Engineering (GHI), RWTH Aachen University, Aachen, Germany; 5 Department of Surface and Plasma Science, Charles University, Prague, Czech Republic; 6 Central Institute for Engineering, Electronics and Analytics (ZEA-3), Forschungszentrum Juelich GmbH, Juelich, Germany; 7 Institute for Electronic Materials (IWE 2), RWTH Aachen University, Aachen, Germany; 8 Juelich-Aachen Research Alliance (JARA-Energy), Juelich, Germany

**Keywords:** Nanoparticles, Fuel cells

## Abstract

Nanostructured composite electrode materials play a major role in the fields of catalysis and electrochemistry. The self-assembly of metallic nanoparticles on oxide supports via metal exsolution relies on the transport of reducible dopants towards the perovskite surface to provide accessible catalytic centres at the solid–gas interface. At surfaces and interfaces, however, strong electrostatic gradients and space charges typically control the properties of oxides. Here we reveal that the nature of the surface–dopant interaction is the main determining factor for the exsolution kinetics of nickel in SrTi_0.9_Nb_0.05_Ni_0.05_O_3–__*δ*_. The electrostatic interaction of dopants with surface space charge regions forming upon thermal oxidation results in strong surface passivation, which manifests in a retarded exsolution response. We furthermore demonstrate the controllability of the exsolution response via engineering of the perovskite surface chemistry. Our findings indicate that tailoring the electrostatic gradients at the perovskite surface is an essential step to improve exsolution-type materials in catalytic converters.

## Abstract (web summary)

The self-assembly of metallic nanoparticles on oxide supports via metal exsolution relies on dopant transport, but strong electrostatic gradients and space charges typically control the properties of surfaces. The surface–dopant interaction is shown to be the main determining factor for the exsolution kinetics of nickel in a perovskite system.

## Main

Innovative concepts for the design of active and stable catalysts can enable key technologies to buffer the intermittency of renewable energy technologies or to produce green hydrogen, using (electro)chemical energy conversion devices such as electrolysers and fuel cells, catalytic membrane reactors or heterogeneous catalysis^1–4^. Here nanocomposites play a crucial role for the development of high-performance energy materials. Metal exsolution processes promise manifold benefits in comparison to conventional methods for the preparation of supported nanoparticles, such as in situ activation and catalyst regeneration by reversible cycling of the process, and have received growing interest in the field of energy materials^5–12^. Perovskite oxides doped with reducible transition metal cations serve as parent compounds for the fabrication of self-assembled, uniformly distributed and highly dispersed metallic nanoparticles on oxide supports. A simple reducing treatment is applied to induce the growth of nanoparticles at the perovskite surface, nanoparticles that are formed from dopant cations. Despite the simplicity of nanocomposite synthesis via the exsolution route, the mechanisms that underpin the metal exsolution process have yet to be resolved and are controversially discussed within the field. The nucleation of metallic nanoparticles is driven by the change in Gibbs free energy Δ*G* associated with the transition from the respective metal oxide to the metallic state at low oxygen partial pressure^5,13^. However, the transport of dopants towards the surface requires a physical or chemical gradient to favour their emergence at the oxide surface rather than in the bulk, where multiple physico-chemical gradients may be involved in modulating the net mass transport towards the perovskite surface (for example, the difference in the surface versus bulk energy or concentration gradients). Although the surface properties of functional oxides are widely mediated by space charge, which is known to dictate the ionic mass transport in many functional oxides^14–22^, space charge has been neglected entirely in the proposed transport mechanisms of exsolution. Hence the interaction of the exsolving species with the energy landscape and the electrostatic field landscape at the surface remains unexplored. Here we present experimental evidence that space charge regions (SCRs) formed at the perovskite surface during thermal oxidation and thermal reduction play a critical role for the exsolution kinetics.

## Surface and bulk exsolution

**Fig. 1:**
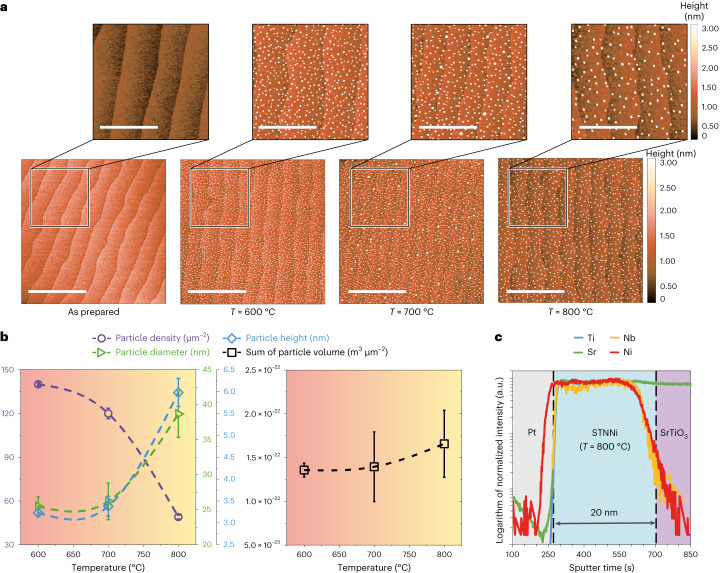
Characterization of STNNi thin films deposited on SrTiO_3_. The 20-nm-thick STNNi thin films were deposited on (001) SrTiO_3_ single crystal substrates and have been thermally reduced at different temperatures (4% H_2_/Ar, *t* = 30 h). **a**, Comparison of the surface morphology in the as-prepared state and after reducing treatment at *T* = 600 °C, *T* = 700 °C and *T* = 800 °C. The atomic force microscopy scan size is 5 × 5 µm^2^ and scale bars are 2 µm (bottom), or 2 × 2 µm^2^ and scale bars are 1 µm (top). Decorations of the surface by exsolved nanoparticles are visible. **b**, Nanoparticle properties determined based on the topological data showing mean values of the particle density, the median particle height and the median particle diameter (±s.d.), each obtained on the basis of three 5 × 5 µm^2^ scans. While the particle density decreases, particle height and particle diameter increase with increasing temperature (left). The mean sum of the volume of the exsolved particles (±s.d.), however, is similar after thermal reduction at different temperatures (right). Note that only the volume of the part of the particles penetrating the surface is detected. **c**, Cation distribution determined by SIMS profiling of a STNNi sample covered with 30 nm of evaporated platinum after reduction at *T* = 800 °C. Nickel accumulation can be observed at the surface, while the concentration appears to remain constant in the thin-film bulk. Dashed lines in **b** serve as a guide for the eye. Dashed lines in **c** denote the interface between the Pt and thin film as well as the interface between the thin film and the substrate. The depth profiles of the different cations in **c** were shifted along the *y* axis for clarity.

The exsolution response of nickel is studied based on well-defined (001)-oriented SrTi_0.9_Nb_0.05_Ni_0.05_O_3–__*δ*_ (STNNi) epitaxial thin films (compare with Supplementary Fig. 1). This approach mimics the surfaces of exsolution-active perovskite materials on a single grain level, while at the same time providing atomically smooth perovskite surfaces and well-defined probing geometries^23^. After a reducing thermal treatment, finely structured decorations are visible at the crystal surface (Fig. 1a). Depending on the applied temperature, a variation in the nanoparticle density and the nanoparticle dimensions is evident (Fig. 1b, left). Interestingly, however, there is no statistically significant difference in the total volume of all nanoparticles after reduction at different temperatures (Fig. 1b, right). Instead, our findings indicate the nucleation of a fixed volume of Ni on the surface and the subsequent nanoparticle growth predominantly by Ostwald ripening. If metal exsolution is considered to be driven by the material striving towards thermodynamic equilibrium and to be based on bulk defect-chemical reactions, the amount of exsolved metal would be expected to be temperature dependent. High temperatures should result in accelerated mass transport and thus a stronger exsolution response that manifests in a larger total volume of exsolved Ni present at the perovskite surface. Alternatively, an equal total nanoparticle volume would be expected only if all Ni dopants that are accommodated within the perovskite parent oxide were exsolved to the surface during thermal reduction at different temperatures.

To evaluate the concentration profile of nickel within the thin film after exsolution, depth profiling by secondary ion mass spectrometry (SIMS) is applied after capping the thin films with platinum to avoid initial sputtering artefacts. Crossing the platinum-to-STNNi interface, a delay between the increase of the Ni signal and the signal of the host cations (Sr, Ti) as well as Nb dopants becomes apparent (Fig. 1c). The early increase of the Ni signal corresponds to the accumulation of nickel at the surface, which is an indicator of Ni exsolution. However, no considerable changes in the Ni distribution across the thickness of the thin film are visible in comparison to the as-prepared sample (Supplementary Fig. 2).

In particular, no depletion of Ni in the film and no gradient in the Ni content is detected by SIMS, where comparable Ni depth profiles are detected by SIMS after reduction at different temperatures (Supplementary Fig. 2). These findings may indicate a limitation of the dopant transport to the surface region in this material system, as shown in other recent studies^24,25^, where higher temperatures lead to the coalescence of the nanoparticles. The exsolution of dopants from larger length scales to the perovskite surface may be competing with the nucleation of nanoparticles within the oxide bulk and hence may be less substantial. Here considerable amounts of Ni dopant may remain immobilized in the thin-film bulk in the form of buried metallic nanoparticles^23,26,27^, in line with our SIMS analysis. Hence, probably only a small fraction of the Ni dopant present in the as-prepared STNNi thin films is exsolved to the surface.

## Probing surface space charge upon exsolution

To resolve the redox response at the STNNi surface, the chemical and electronic processes are probed by near-ambient pressure X-ray photoelectron spectroscopy (NAP-XPS). The spectroscopic signature is monitored in situ during the exsolution process at a constant sample temperature, while the oxygen chemical potential is controlled by the supply of oxygen and hydrogen gas. As discussed above, metal exsolution appears to be mostly limited to the surface region of the perovskite thin film (compare with Fig. 1), which compares well with the typical screening lengths of SCRs in highly doped oxides^28,29^. To investigate the formation of SCRs and their influence on metal exsolution, XPS core-level spectra are recorded to compare two different measurement protocols.

**Fig. 2:**
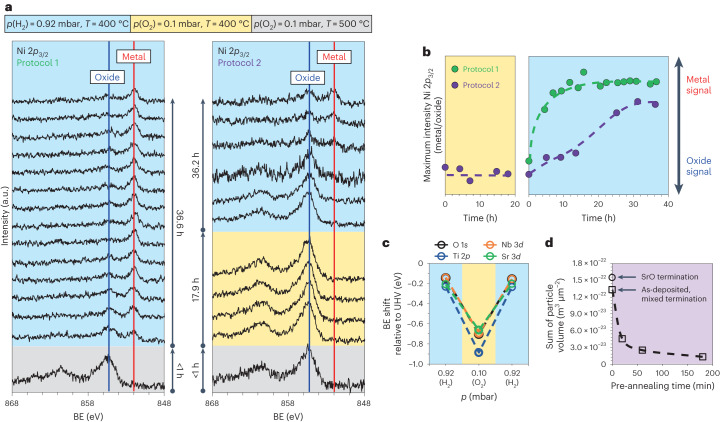
Analysis of the interplay between redox chemistry of STNNi and nickel exsolution. **a**, In situ analysis of the Ni 2*p*_3/2_ core-level spectrum by NAP-XPS during reducing and oxidizing sample treatments at elevated temperatures using two different annealing protocols. Rapid formation of a metallic signature can be observed during reduction (protocol 1). By contrast, oxidizing pretreatment of the STNNi thin films results in a considerably delayed redox response, that is, a delayed formation of metallic nanoparticles (passivation effect), at the perovskite surface (protocol 2). The BE values of all spectra were corrected to the Ti 2*p* position, and the intensity was normalized to the prepeak region. **b**, Illustration of the exsolution dynamics during protocol 1 and protocol 2 based on the signal ratio of the metal and oxide features. The evolution of a metallic Ni signal is strongly delayed after the oxidizing pre-annealing performed during protocol 2. **c**, Based on shifts in the BE relative to ultra-high vacuum (UHV) conditions, the formation of a surface SCR can be observed. The space charge potential is different for oxidizing and reducing conditions as it depends on the respective electric and ionic reconstruction in the surface region. **d**, Ex situ studies of the surface passivation effect due to oxidizing pre-annealing show that the delayed exsolution is directly correlated to a reduced particle volume as determined by microscopic investigations. The sample pieces were pre-annealed under oxidizing conditions for *t* = 20 min, *t* = 60 min and *t* = 180 min (*p*(O_2_) = 0.108 mbar, *T* = 400 °C) and compared to an as-prepared sample and a sample with an intentional SrO termination layer after thermal reduction in the same process step (4% H_2_/Ar, 400 °C, *t* = 5 h). Dashed lines serve as guides for the eye. Sample conditions are denoted by the colour code (blue, yellow and grey shading) at the top of **a**.

First, the redox chemistry is investigated during a fast introduction of hydrogen into the XPS chamber (Fig. 2a, protocol 1). In a second measurement, the influence of an initial oxidizing thermal treatment on the exsolution response is studied, which is performed prior to the reducing annealing step (Fig. 2a, protocol 2). We pay special attention to the spectroscopic changes visible in the Ni 2*p* region to track the evolution of a metallic Ni signature indicative of the formation of metal nanoparticles, where we qualitatively follow the chemical state of nickel dopants based on the main peak of the Ni 2*p*_3/2_ core-level signal.

For the as-prepared state, a peak centred at a binding energy (BE) of ~855.6 eV is visible (oxidized state) while the main peak of the metallic Ni 2*p*_3/2_ state is centred at a BE of ~851.9 eV. As shown in Fig. 2a,b (protocol 1), the introduction of hydrogen results in a rapid formation of metallic Ni species at the thin-film surface, as indicated by the fast evolution of a metallic signal. The coexistence of Ni in the oxidized state is evident based on the presence of a (remaining) oxide signal of considerably reduced intensity as compared to the pristine state. Furthermore, over time, the metallic signal slightly increases while the oxide signal in turn decreases during the reducing treatment. By contrast, in situ spectroscopy reveals no considerable changes in the Ni surface chemistry during the initial oxidizing treatment of STNNi (Fig. 2a,b, protocol 2). After holding the measurement conditions constant for a time *t* of about 17.9 h, hydrogen is introduced into the chamber to induce the exsolution process. Interestingly, the rapid exsolution response observed during protocol 1 remains absent for the oxidized sample. More precisely, the exsolution of metallic nanoparticles to the perovskite surface is strongly retarded. While a rapid evolution of a metallic signal was observed immediately after the introduction of hydrogen during protocol 1 (after *t* ≈ 4.5 h), indications of the formation of a metallic signature during protocol 2 are first visible after *t* ≈ 25 hours of reduction (Fig. 2b).

Comparing the measurement protocols presented above, a clear passivation effect of the oxidizing pretreatment that delays the exsolution response is apparent. Complementary morphological studies of STNNi samples were performed ex situ, after an oxidizing pre-annealing treatment at temperature *T* = 400 °C and oxygen pressure *p*(O_2_) = 0.108 mbar, conditions comparable to the conditions present during the in situ NAP-XPS investigations. With an increasing duration of the oxidizing pre-annealing (*t* = 20 min, *t* = 60 min and *t* = 180 min), the subsequent reduction of the thin-film samples at *T* = 400 °C under 4% H_2_/Ar gas flow for *t* = 5 h results in striking differences of the exsolved particle volume. As shown in Fig. 2d, the particle volume strongly decreases for samples that have been oxidized before the reducing treatment (atomic force microscopy scans are in Supplementary Fig. 3a). Here, even short pre-oxidation times have a large impact on the exsolution response of nickel induced during a comparably long reducing treatment of five hours. Notably, the low pre-oxidation temperatures kinetically suppress any substantial changes of the crystallographic properties of the bulk material, clearly indicating a surface process as the origin of the observed passivation effect and in line with the NAP-XPS results. Supplementary Note 3 contains detailed discussions of the crystal structure, surface morphology and stoichiometry. Moreover, we can exclude that the surface passivation effect, which results in a decrease of the exsolution volume by up to one order of magnitude, is a result of an altered termination layer of the perovskite. As Fig. 2d shows, a similar total nanoparticle volume is detected for STNNi with a native, mixed termination layer (the as-deposited sample) and for STNNi with an intentional SrO termination layer, which was fabricated by sequential epitaxy (compare with Supplementary Fig. 5).

Apart from the clear changes in the chemical state of Ni, more subtle changes can be observed in the signatures of the other elements, that is, in the Ti 2*p*, Sr 3*d*, Nb 3*d* and O 1*s* core-level spectra discussed in Supplementary Note 5. Prominent and uniform shifts in the BE values of the core-level spectra of all elements, as well as a broadening of the signals, are evident in the different gas atmospheres. The shift in BE is plotted in reference to the peak position recorded in an ultra-high vacuum (at room temperature) in Fig. 2c for oxidizing and reducing conditions. The magnitude of the shift in BE depends on the ambient reducing and oxidizing conditions and was shown to be related to the presence of SCRs at the surface^30–32^. In addition, reversible peak broadening, observed for the XPS core-level spectra, may be directly related to the formation of SCRs at the perovskite surface^33,34^, while different contributions, for example the evolution of a minor SrO or Sr(OH)_2_ surface phase and an altered scattering behaviour of photoelectrons, need to be considered (compare with Supplementary Fig. 6). The formation of surface SCRs is related to the inherent defect chemistry and the respective redox response of the material under different oxygen chemical potentials^18,30,35,36^. Notably, the timescale of the retarded exsolution response after the oxidizing pre-annealing is very similar to the dynamic response of the space charge layer, detected for Nb-doped SrTiO_3_ by electrical measurements^31^. Our observations hence indicate that the kinetics of Ni exsolution in STNNi are considerably affected by the presence of surface space charge. Notably, repeated cycling results in reversible and reproducible BE shifts accompanied by respective changes in the width of the photoemission signals (compare with Supplementary Fig. 6b–d)^30,36,37^.

## The role of surface space charge for metal exsolution

To understand the nature of the surface SCRs under oxidizing and reducing conditions and their influence on the exsolution process, the specific structural characteristics of the present material system have to be considered. As we have shown in our previous work, nickel dopants in STNNi thin films tend to accumulate in dopant-rich nanostructures rather than dissolve homogeneously within the host lattice^23^. The Ni-rich clusters serve as centres for the nucleation of metallic nanoparticles in the thin-film bulk. Thus, the host lattice is predominantly Nb doped and follows the redox chemistry of donor-doped strontium titanate.

**Fig. 3:**
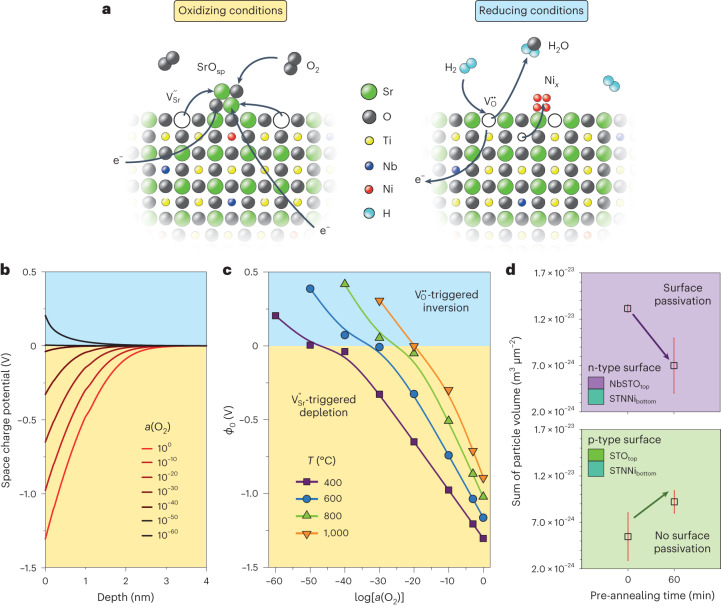
Schematic illustration of the redox-dependent defect formation at the STNNi surface, simulated potential profiles for different oxygen activities and temperatures and epitaxial engineering of the surface defect chemistry for tailoring the metal exsolution response. **a**, Simplified schematic illustration of the redox response of the STNNi host lattice and its impact on the diffusion of charged dopants (such as ${{{\mathrm{Ni}}}}_{{{\mathrm{Ti}}}}^{{\prime}}$) to the surface. Negatively charged Sr vacancies are formed under oxidizing conditions, which are kinetically trapped in the near-surface region, and hence a negative space charge potential is established. A positive space charge potential may be formed under highly reducing conditions by the generation of oxygen vacancies energetically trapped in the near-surface region. Kröger–Vink notation is used in the sketch; e^–^, electron. In SrO_sp_, sp is the surface phase. **b**, Simulated potential profiles of SCRs depending on the oxygen activity as determined by finite-element electrostatic space charge simulations for a temperature of *T* = 400 °C. Negative surface space charge potentials result from thermal annealing in a wide range of oxygen activities. The magnitude of the space charge potential decreases with decreasing oxygen activity. An inversion of the surface space charge towards a positive potential can be observed at very low oxygen activities. **c**, Dependence of the space charge potential (*ϕ*_0_) on the temperature and the oxygen activity. The temperature necessary to induce an inversion of the space charge potential increases with increasing oxygen activity. **d**, Comparison of the mean sum of particle volume (±s.d.) of STNNi (STNNi_bottom_) with a four-monolayer-thick top layer of donor-doped Nb:SrTiO_3_ (NbSTO_top_) or undoped SrTiO_3_ (STO_top_; naturally acceptor doped by impurities). After initial thermal oxidation (*p*(O_2_) = 0.108 mbar, *T* = 400 °C, *t* = 60 min), the samples are thermally reduced (4% H_2_/Ar, *T* = 600 °C, *t* = 5 h). A clear influence of the redox chemistry of the top layer material on the exsolution response is apparent. The nanoparticle volume is determined based on the topological data, showing averaged values obtained from three 2 × 2 µm^2^ scans for each condition.

For Nb-doped SrTiO_3_, surface SCRs can be of two different kinds (Fig. 3a). Oxidizing treatment results in the annihilation of surface-near oxygen vacancies present in the perovskite host lattice. Additionally, to compensate for the charge of donor-type dopants such as niobium, SrTiO_3_ tends to form strontium vacancies, as mediated by the partial Schottky equilibrium^30,35,36,38–40^. Due to sluggish kinetics, these negatively charged defects are kinetically trapped in the surface region of the perovskite and form a negative net surface charge. The spatial accumulation of negative charge results in the formation of space charge with a negative space charge potential. As a consequence, a negative space charge potential is established at the perovskite surface (Fig. 3a, left), consistent with rigid shifts in the BE of the core-level spectra recorded by NAP-XPS towards lower values. This results in a repulsive interaction for negatively charged species, such as mobile acceptors (for example, Ni in SrTiO_3_), due to the gradient of the electrostatic potential between the surface and bulk. The negative potential hence reflects an energy barrier with blocking character for negative charges. By contrast, a thermal treatment at highly reducing conditions may widely suppress the formation of Sr vacancies while oxygen vacancies are generated, energetically trapped and thus predominantly present at the surface of the perovskite oxide (Fig. 3a, right)^18,21,35,41^. Hence a negative space charge potential is diminished during a reducing treatment^39,42^, and eventually, even an inversion of the electrostatic potential gradient may occur^35^.

In Fig. 3b, we show simulated potential profiles of near-surface SCRs depending on the oxygen activity as determined by finite-element electrostatic space charge simulations, which cover a wide range between oxidizing and highly reducing conditions. The kinetics of SCR formation is determined by the species of lowest mobility, that is, diffusion of Sr vacancies in oxidizing conditions, which at low temperature is limited solely to the surface. At *T* = 400 °C the formation of a negative SCR occurs during annealing at oxygen activities down to *a*(O_2_) ≈ 10^–40^, while the inversion towards a positive SCR requires highly reducing conditions. At an oxygen activity of *a*(O_2_) ≈ 10^–50^ (comparable to the oxygen activity present in hydrogen during our experiments), the negatively charged blocking potential is fully absent. A further decrease of the oxygen activity results in the establishment of a positive potential at the perovskite surface. Notably the width of the SCRs is comparably narrow at different temperatures since the screening length is mainly determined by the (niobium) doping concentration. The inversion of the space charge potential observed in Fig. 3b,c indicates that only at extremely low oxygen activity, the oxygen chemistry of STNNi governs the space charge and may even lead to an accelerated exsolution response, while the A-site cation chemistry governs the kinetics in more oxidizing conditions.

Since the redox response is determined by the redox chemistry of the topmost surface of the perovskite and particularly the dopant concentration, slight modifications of the surface redox chemistry should enable tailoring of the exsolution behaviour. For this purpose, epitaxial thin-film samples are deposited in a so-called stack geometry, combining epitaxial STNNi with either a top layer of SrTi_0.95_Nb_0.05_O_3–*δ*_ (donor doped) or a top layer of nominally undoped SrTiO_3_ (typically naturally acceptor doped by impurities^18,21,36^). In this configuration, a lack of donor dopants close to the surface will suppress Sr vacancy formation and thus the formation of a blocking surface depletion layer^43^. Only four monolayers of each Ni-free material are epitaxially deposited on top of the exsolution-active STNNi bottom layer (20 nm), predominantly changing the surface redox behaviour. The exsolution of Ni dopants through the originally Ni-free top layer is visible after the thermal reducing treatment (compare with Supplementary Fig. 7). However, the passivation effect for the donor-doped surface becomes apparent in the form of a reduced volume of exsolved Ni nanoparticles (Fig. 3d). An oxidizing treatment of the stack sample with an undoped top layer, however, does not result in surface passivation. Here, the SrTiO_3_ top layer determines the surface redox response of the stack sample.

**Fig. 4:**
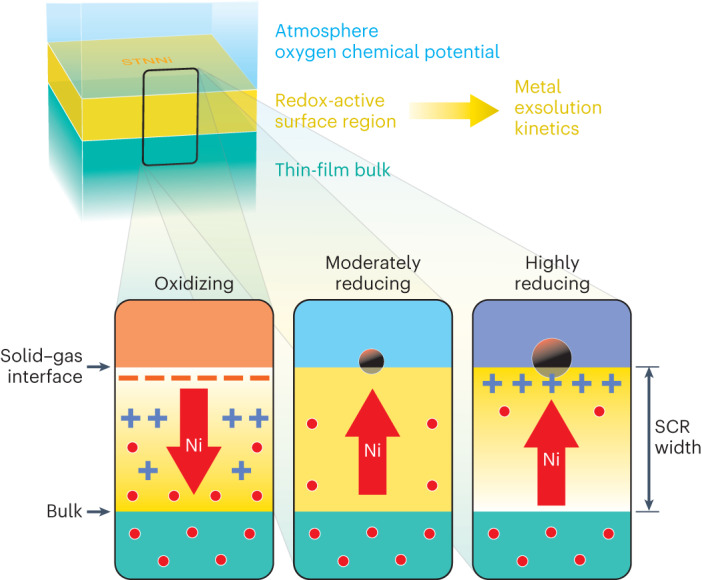
Influence of surface SCRs on metal exsolution kinetics at different oxygen chemical potentials, comparing oxidizing, moderately reducing and highly reducing conditions. In oxidizing conditions, a negative space charge potential is induced at the surface, which results in a repulsive interaction with Ni dopants of relative negative charge compared to the host cations. The magnitude of the space charge potential decreases with decreasing oxygen activity, that is, under reducing conditions, and allows for the exsolution of Ni to the surface region, which serves as a sink of Ni dopants forming metallic nanoparticles. In consequence, a concentration gradient of Ni dopants evolves, which further promotes the exsolution of dopants to the surface. At highly reducing conditions, an inversion of the SCR towards a positive surface potential may promote the exsolution of dopants by attractive electrostatic interactions. The presence of near-surface SCRs is strongly entangled with the surface redox chemistry of the perovskite host lattice and widely dictates the exsolution kinetics. The electrostatic gradient of the SCR, critical for the release of dopants to the perovskite surface, is mainly determined by oxygen activity and temperature during thermal treatment.

SCRs are well-known to influence a wide range of properties in oxides and particularly in functional ceramics^14–22^. For instance, electrostatic interactions of dopants with surface SCRs are known to promote the segregation of dopants during thermal treatment^19^. SCRs also determine the grain boundary conductivity in ionically conducting ceramics^44^. Here, the nature of the potential gradient, the charge of dopants and the defect chemistry of the host lattice are crucial for the electrostatic interactions. Nickel dopants exhibit a negative charge relative to the B-site cations of the host lattice Ti (IV) and Nb (V). Occupying the B site of the perovskite structure, nickel dopants are expected to exhibit the nominal charge (III), while Ni dopants present in the form of NiO_*x*_ nanostructures (with *x* ≈ 1)^23^ are expected to have the nominal charge (II). Thus, at elevated temperatures and under oxidizing conditions, repulsive interactions of diffusing Ni ions with a negative surface SCR can explain the observed surface passivation effect, resulting in a retarded exsolution response (Fig. 4, left). Such conditions are typically present during the synthesis of exsolution-active perovskites, with dopants accommodated within the oxide. The decrease of the negative space charge potential under reducing conditions results in a reduction of the blocking surface potential (Fig. 4, centre). The solubility of Ni in the host matrix is considerably decreased at low oxygen pressures, resulting in the phase transition towards metallic nickel. Diffusive dopant transport of Ni ions in the surface region leads to accumulation of Ni dopants in the form of nanoparticles at the surface. The reduction to metallic Ni at the surface causes a concentration gradient of nickel ions in the near-surface region, since the surface acts as a sink for Ni ions. In this way, the presence and strength of SCRs at the surface can delay the exsolution response of the oxide.

An inversion of the space charge potential towards a positive surface charge under highly reducing conditions may even lead to attractive electrostatic interactions of Ni dopants with the surface (Fig. 4, right panel). A positive surface charge region generated during reducing annealing and based on the enrichment of oxygen vacancies in the surface region consequently may serve as a gradient to further promote exsolution to the perovskite surface and to considerably accelerate the kinetics of metal exsolution. However, such extremely low oxygen activities are scarcely accessible under technically relevant conditions, and the exsolution of acceptor-type dopants is expected to be mainly induced by the decrease of the blocking negative surface potential. Notably, thermodynamically derived parameters such as the enthalpy of oxygen vacancy formation will influence the magnitude of the surface space charge potential and therefore may be a suitable lever to drive the surface potential into inversion and to accelerate metal exsolution processes.

Besides electrostatic interactions, SCRs may influence the exsolution response due to the decrease of oxygen mobility in the near-surface region associated with the redox chemistry of the host lattice. The oxygen vacancy concentration is typically low in primarily donor-doped SrTiO_3_. Consequently, the oxygen transport is rather slow, in particular after oxidizing treatment accompanied by the annihilation of oxygen vacancies. The kinetics of oxygen exchange—fundamentally driving the exsolution process via the equilibration of the oxygen chemical potential in the material and the external atmosphere—is hence decreased when a negative SCR is present in the surface region. The surface passivation is coupled to both cation redox chemistry and oxygen chemistry, which are strongly entangled via space charge formation modulating the mass transfer dynamics during metal exsolution. The space charge effect, hence, may not be solely based on electrostatic interactions, but in addition on the basis of the underlying redistribution of cationic and anionic defects with influence on the diffusion of reducible cations^45^ and oxygen anions^46^ within the confined SCR. Subtle changes in the surface chemistry due to the enrichment of Sr species resulting from the introduction of Sr vacancies at the perovskite surface in the course of space charge formation may furthermore result in minor changes in the nucleation behaviour during metal exsolution. The surface passivation effect, however, cannot be explained by a mere change in the surface chemistry towards a SrO termination layer (compare with Supplementary Fig. 5). Space charge hence determines the mass transport dynamics of exsolving metal species across the solid–gas interface, while additional parameters such as the surface energy of the metal nanoparticles on the surface may control the subsequent size and distribution of the exsolved surface nanoparticles^47^.

Space charge formation at grain boundaries may also strongly influence the exsolution behaviour in ceramic materials^18^. Here, space charge interactions may be associated with the preferential nucleation of nanoparticles along grain boundaries as reported in the literature^48^. Since space charge formation heavily depends on the local defect formation enthalpies close to the surface, orientation‐dependent chemical composition and surface reconstructions^49^ may influence the nature of surface SCRs formed at different crystallographic facets of powder grains^18,47,50^ and hence need to be investigated to improve the understanding of metal exsolution processes in functional ceramics. Since the enthalpy of formation for A-site vacancies in perovskites strongly influences the formation of SCRs, one can hypothesize that the chemical nature of the A-site ions strongly influences the exsolution of B-site dopants. Specifically, the well-documented enhancement of exsolution phenomena by A-site deficiency^5^ may, at least in part, be related to modifications of the surface defect concentrations (and hence SCRs) by the introduction of high concentrations of A-site vacancies in the bulk material. Furthermore, the reversibility, that is, the switchability, of exsolution processes by alternating the reduction and re-oxidation of exsolution catalysts may be limited due to the presence of SCRs. Rapid formation of a near-surface SCR under oxidizing conditions may alter the kinetics of the indiffusion of Ni dopants. This is of great importance for the application of exsolution-active materials in catalytic converters, which aims to make use of the switchability of the catalysts based on the dynamic exsolution and dissolution of nanoparticles, and which requires a fast response to the chemical environment^11^. Here the influence of surface space charge on the exsolution kinetics needs to be taken into account.

Directly associated with the formation of a negative SCR at perovskite surfaces, distinct passivation effects can occur under oxidizing conditions. The mechanistic understanding of the transport of dopants to the perovskite surface then derives from the electrostatic gradients present at the surface. These are determined by the redox chemistry of the perovskite surface, and dictate the kinetics of metal exsolution processes. Surface passivation manifests in a substantially retarded exsolution response. Under reducing conditions, the equilibrium of the redox reaction relaxes over time and the blocking potential decreases, allowing for the exsolution of acceptor-type dopants to the surface after an initial delay. By contrast, an inversion of the SCR towards a positive surface potential under strongly reducing conditions may even promote the exsolution kinetics and hence the formation of metallic nanoparticles at perovskite surfaces.

Tailoring the properties of surface SCRs opens new strategies for the rational design of exsolution materials. Here, control of the respective screening length via co-doping of the host lattice with elements that are stable under reducing conditions may allow one to adjust the dynamics of the exsolution response and the exsolution depth. As was demonstrated, slight modifications of the redox chemistry of the topmost surface can be applied to heavily modify the exsolution response of doped perovskite oxides, which provides new perspectives for the control of metal exsolution processes by surface and interface engineering.

## Methods

### Epitaxy

Epitaxial STNNi thin films of 20 nm and 50 nm thickness were deposited with monolayer precision using pulsed laser deposition controlled by reflection high-energy electron diffraction (Supplementary Fig. 1a). The experiments typically were performed using thin films of 20 nm thickness. Only when crystallographic properties were investigated (compare Fig. 2d and Supplementary Fig. 3) were thin films of 50 nm thickness employed, to allow for the investigation of a clearly separated thin film and substrate signal in X-ray diffraction. For pulsed laser deposition growth, single crystalline (001) SrTiO_3_ and Nb:SrTiO_3_ substrates (Shinkosha) were applied (Supplementary Fig. 1b). For TiO_2_ termination, the substrates were etched in buffered HF and annealed at *T* = 950 °C under a continuous flow of an O_2_/Ar (20:80) gas mixture for two hours. Nb-doped substrates were used when the analysis required a fully conductive sample. Thin-film growth was performed at a constant backside temperature of *T* = 650 °C using an infrared-diode laser with a wavelength of *λ* = 925 nm for heating. A KrF excimer laser (COMPex 205 F-version, Coherent) operated with a repetition rate of *f* = 5 Hz and with a wavelength of *λ* = 248 nm was applied for the ablation of the ceramic target material. The thin films were deposited at oxygen pressures of *p*(O_2_) = 0.108 mbar and using a laser fluence of *F* = 1.14 J cm^−2^. The thin-film samples were immediately quenched to room temperature after the deposition was completed (no post-annealing). The ceramic STNNi target was prepared by cold isostatic pressing and subsequent sintering of a powder synthesized by the Pechini method. The target-to-substrate distance was *d* = 57 mm.

### Thin-film processing

Thermal treatment under reducing conditions (4% H_2_/Ar) was applied to induce the exsolution process. After the reducing thermal treatment, the thin-film samples were quenched to room temperature under continuous gas flow. An oxidizing pre-annealing was performed in the pulsed laser deposition chamber at an oxygen pressure of *p*(O_2_) = 0.108 mbar and a temperature of *T* = 400 °C using an infrared-diode laser with a wavelength of *λ* = 925 nm for heating.

### Thin-film characterization

The surface morphology was characterized by atomic force microscopy (Cypher, Oxford Instruments Asylum Research), and the particle properties such as nanoparticle density, nanoparticle dimensions (median diameter and median height) and nanoparticle volume were determined from the topological data using Gwyddion 2.52. A lower threshold of 1 nm was applied to analyse the nanoparticle properties, with the zero level set on one of the central perovskite terraces. Crystallographic properties were investigated by X-ray diffraction analysis in the Bragg−Brentano geometry (D8 Discover, Bruker AXS). The diffractometer was equipped with a Goebel mirror, a Cu ${k}_{{\alpha}_{1}}$ monochromator, a Centric Eulerian Cradle, a Lynxeye XE detector and a pinhole adaptor (2 mm in diameter). Depth profiling of the cation distribution (Ti^+^, ^86^Sr^+^, Ni^+^, Nb^+^ and Pt^+^ signals) was performed by time-of-flight secondary ion mass spectrometry (ToF-SIMS_5.NCS, IONTOF) in negative polarity mode after 30 nm of platinum were evaporated at the sample surface.

### In situ spectroscopy

In situ analysis of the exsolution response was performed by custom-designed laboratory-based NAP-XPS (SPECS Surface Nano Analysis, with monochromatized Al *K*_α_ source). To avoid charging effects during in situ spectroscopy, conductive Nb-doped SrTiO_3_ substrates were employed for the epitaxial growth of STNNi. The samples were fixed at the sample holder of the NAP-XPS system using metal strips in order to ensure good electrical and thermal conductivity. Here, the K-type thermocouple was placed in between the sample surface and one of the metal stripes. Sample heating was realized by electron bombardment at the back of the sample stage while the sample was mounted on the NAP manipulator. XPS Ti 2*p*, Sr 3*d*, Ni 2*p*, Nb 3*d*, O 1*s* and C 1*s* core-level spectra were recorded during exposure of the samples to hydrogen or oxygen gas at a constant temperature of *T* = 400 °C after a short heating period of each sample to *T* = 500 °C under an oxygen atmosphere to remove carbon species adsorbed at the surface. The absolute BE scale of all spectra displayed in Fig. 2a was calibrated to the position of the Ti 2*p*_3__/__2_ core-level signal recorded under ultra-high vacuum conditions and at room temperature (BE = 458.4 eV), and the intensity was normalized to the prepeak region. No BE correction was performed for the core-level spectra displayed in Supplementary Fig. 6b,d. Typically, 50 scans were averaged for each Ni 2*p* spectrum shown. In a few cases, a smaller number of scans was used due to slight shifts in the sample position over the long probing durations, which resulted in a decreased signal-to-noise ratio of the signal over time. Notably, the intensity of the Ni 2*p* core-level signals is not quantitative due to the ongoing lateral separation of the material system during nanoparticle formation, which results in continuous changes in the measurement geometry upon metal exsolution. However, the intensity ratio of the metal and oxide signals can be used to monitor the dynamics of nanoparticle formation at the perovskite surface. The core-level spectra were recorded with a pass energy of 20 eV, a step size of 0.05 eV, a dwell time of 200 ms and a spectral resolution of 0.8 eV.

### Finite-element electrostatic space charge simulations

Space charge calculations were based on bulk defect concentrations calculated for SrTiO_3_ via bandgap excitation, oxygen exchange reaction and Nb-dopant concentration. Space charge formation was calculated considering the dedicated surface defect reactions, including an active surface Schottky equilibrium (strontium vacancy formation^48^) as well as a reduced enthalpy of formation for oxygen vacancies in the most near-surface atomic layer^24^. As a result of the varied surface equilibria, surface defect concentrations differed from the expected bulk value, resulting in a redistribution of mobile defects and formation of a surface space charge layer, obeying electrostatic boundary conditions. Electrostatic potential, electric field and local defect concentrations were solved self-consistently using a finite-element routine, with the boundary condition of vanishing space charge in the bulk (global charge neutrality). The *a*(O_2_) dependence was derived from the intrinsic *a*(O_2_) dependence of the defect equilibria. A detailed list of reaction equations and input parameters is given in Supplementary Note 7.

## Online content

Any methods, additional references, Nature Portfolio reporting summaries, source data, extended data, supplementary information, acknowledgements, peer review information; details of author contributions and competing interests; and statements of data and code availability are available at 10.1038/s41563-023-01743-6.

### Supplementary information

Supplementary Information Supplementary Notes 1–7, Figs. 1–7 and Table 1.

## Data Availability

The data supporting the findings of this study are available within the paper and its Supplementary Information file. The experimental data are available at 10.26165/JUELICH-DATA/CYHXRM.
