# Supplementary material for: Space charge governs the kinetics of metal exsolution
Source: Nat Mater. 2024 Jan 2;23(3):406–13. doi: 10.1038/s41563-023-01743-6 (PMC10917682; doi:10.1038/s41563-023-01743-6)
Supplement: Supplementary file 1 — Supplementary Notes 1–7, Figs. 1–7 and Table 1. [file 41563_2023_1743_MOESM1_ESM.pdf]

nature materials

Article

https://doi.org/10.1038/s41563-023-01743-6

Space charge governs the kinetics of metal exsolution

In the format provided by the authors and unedited

Table of contents

Supplementary Note 1: Thin film fabrication	S-2
Supplementary Note 2: Cation depth-profiling	S-2
Supplementary Note 3: Oxidizing pre-annealing	S-3
Supplementary Note 4: Epitaxial control of the surface termination	S-6
Supplementary Note 5: Redox response of STNNi	S-7
Supplementary Note 6: Surface engineering of the surface redox chemistry	S-9
Supplementary Note 7: Finite-element electrostatic space charge simulations	S-11
References	S-14

Supplementary Note 1: Thin film fabrication

The thin films are epitaxially deposited in layer-by-layer growth mode by pulsed laser deposition as revealed by *in-situ* reflection high-energy electron diffraction (RHEED)-monitoring. Each oscillation visible in Fig. S1a refers to the deposition of one monolayer of STNNi. The thin film material is deposited in [001]-orientation with a sharp thin film-to-substrate interface indicated by distinct KIESSIG oscillations in the vicinity of the thin film X-ray diffraction peak (Fig. S1b).

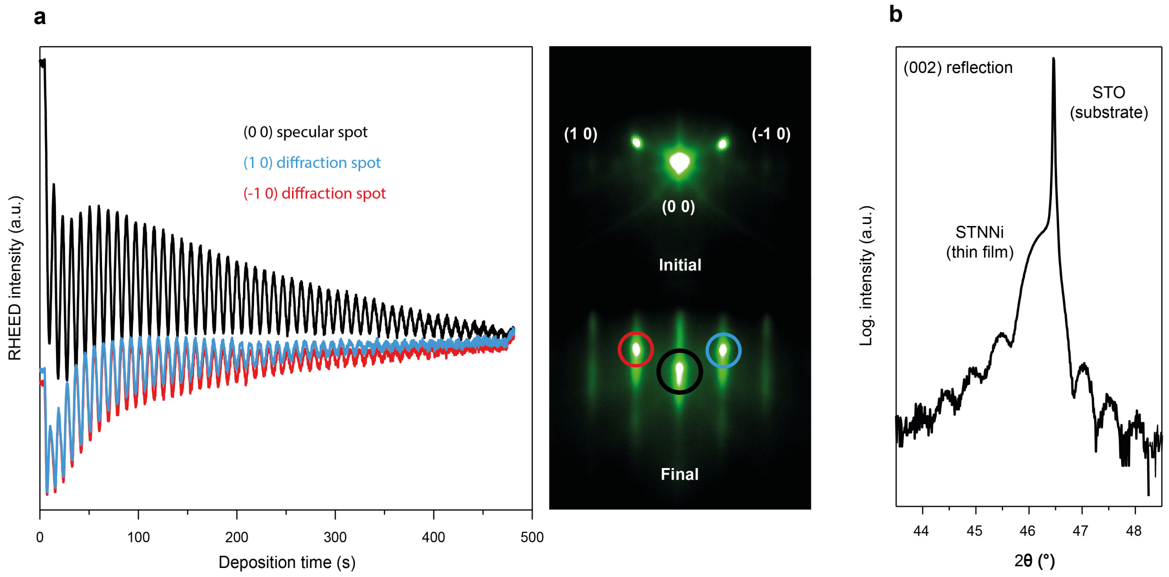

Fig. S1| (a) RHEED-controlled pulsed laser deposition of epitaxial STNNi thin films on (001) $SrTiO_3$. The intensity evolution of the (0 0) specular spot and (1 0), (-1 0) diffraction spots is used to monitor the layer-by-layer thin film growth. Two-dimensional RHEED patterns are evident in the initial state (substrate surface) and the final growth state (thin film surface). (b) X-ray diffraction analysis of a representative STNNi thin film with a thickness of 20 nm around the (002) diffraction peak in 2θ-ω geometry. The sharp peak of the $SrTiO_3$ substrate is partly superimposed by the (naturally) broadened thin film peak, visible in form of a pronounced shoulder at lower diffraction angles. KIESSIG fringes are visible in the vicinity of the main reflections.

Supplementary Note 2: Cation depth-profiling

The cation distribution at the thin film surface and within the thin film bulk is probed by secondary ion mass spectrometry after the thin film samples were capped by platinum to avoid initial sputter artefacts at the surface (Fig. S2). After a reducing thermal treatment, the accumulation of nickel at the thin film surface is visible based on the delay between the increase in the Ni-signal and the increase of the signal of the host cations (Sr, Ti) as well as the redox stable Nb-dopants.

As can be seen, the intensity profiles of the top layer cations exhibit a tailing feature when crossing the interfaces of a top layer (*e.g.* Pt) to the respective bottom layer (*e.g.* STNNi), which results from minor sputtering events at the inner walls of the slightly cone shaped sputter crater (as illustrated in Fig. S2a). Thus, the position of the platinum-to-thin film interface is determined based on the steep increase in the signals of the host cations, which do not suffer from this effect during the first penetration of the thin film surface (intensity jump without tailing feature). For comparison, a modified plot of the sputter profiles obtained from an STNNi thin film after reducing thermal treatment at $T = 800°C$ is shown which includes the sputter profile of platinum (Fig. S2a, right panel, intensity drop of Pt signal with tailing feature)..

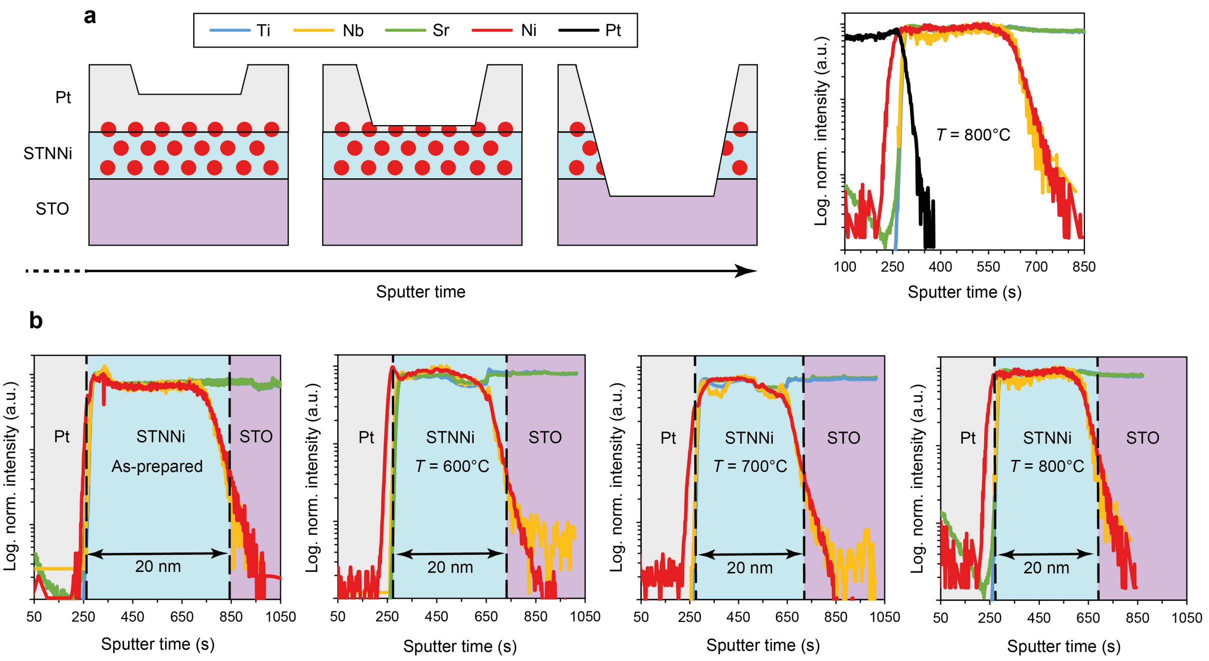

Fig. S2| Depth-profiling of the cation distribution over the thin film thickness by secondary ion mass spectrometry comparing STNNi samples in the as-prepared and reduced state (cut from the same thin film) after reducing thermal treatment at different temperatures (4% H_2/Ar, t = 30h). (a) Schematic sketch of the sputter process. An early increase of the Ni signal and respectively a delayed increase of the Sr, Ti and Nb signals indicates accumulation of Ni at the thin film surface after the reducing thermal treatment (left). Sputter profile obtained from STNNi after reducing treatment at T = 800°C (shown in Fig. 1c in the main manuscript), including the sputter profile of Pt (right panel) (b) A nickel accumulation zone of similar thickness is visible after reduction at different temperatures of T = 600°C, T = 700°C and T = 800°C. The samples are equal to Fig. 1 of the main manuscript.

To determine the position of the thin film-to-substrate interface however, one relies on the Ni- and Nb-signals (intensity drop with tailing feature). The interface positions are determined to be located at the point where the intensity decreased to about half of the initial signal (on the log scale, typically corresponding to a drop of relative intensity by a factor of 10-100 with respect to the maximum). The sputter times may slightly vary due to small variations in the sputter area and small differences in the sputter behavior as a result of the different sample processing (*e.g.* oxide *vs.* metallic state of Ni). Please note that the accumulation width is estimated based on the sputter rate obtained from the oxide thin film and hence potential deviations in the sputter rate of the metallic nanoparticles were neglected.

Supplementary Note 3: Oxidizing pre-annealing

If the samples are pre-annealed in oxidizing conditions, considerable differences in the exsolved Ni volume are detected (after reducing thermal treatment) by investigations of the surface morphology (Fig. S3a), which reflects the retarding influence of a negative (blocking) surface potential established under oxidizing conditions. X-ray diffraction analysis reveals no changes in the thin film diffraction pattern after the oxidizing annealing, which is mainly determined by the doping level of the host lattice and the defect structure (Fig. S3b).[1,2] Typically, a reducing thermal treatment at high temperatures (> 600°C) results in a relaxation of the host lattice due to exsolution of Ni-dopants to the surface and the formation of metallic nanoparticles in the oxide bulk.

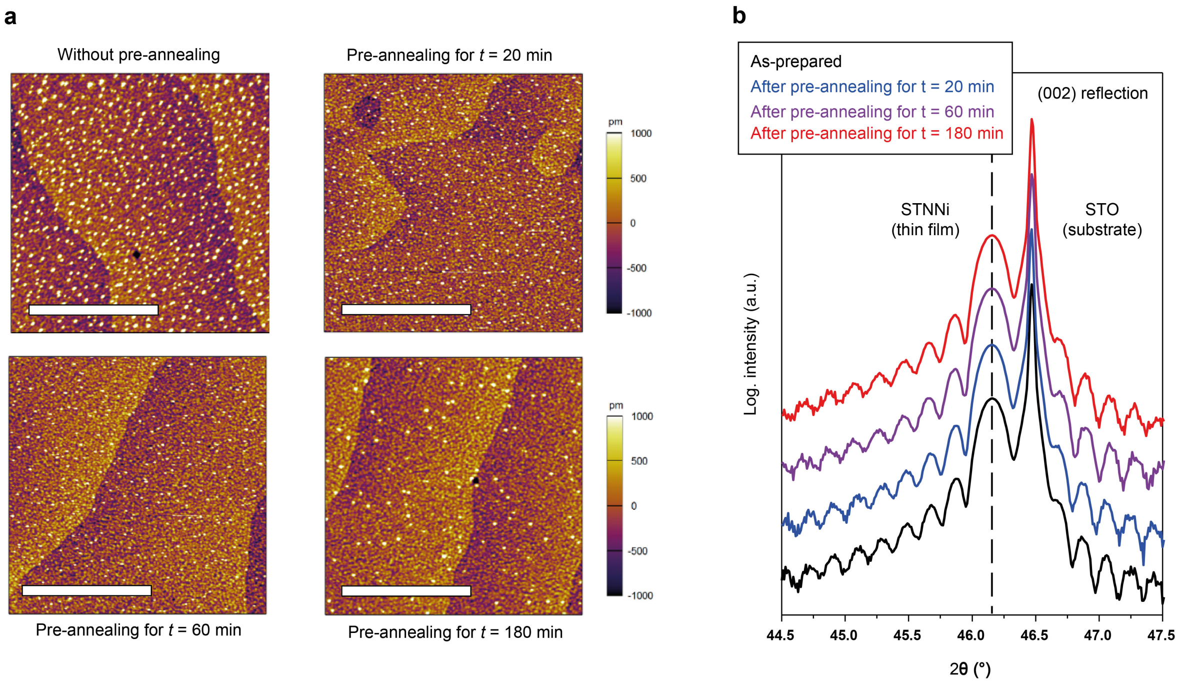

Fig. S3| (a) Surface morphology of STNNi samples which were reduced (reducing conditions: 4% H_2/Ar, T = 400°C, t = 5 h) after oxidizing pre-annealing (oxidizing conditions: $p(O_2)$= 0.108 mbar, T = 400°C). The AFM scan size is 2x2 μm^2 and scale bars 1 μm respectively. After oxidizing treatment, the volume of nickel particles exsolved to the surface upon reducing treatment is significantly decreased. (b) X-ray diffractograms obtained from the oxidized thin film samples. No changes in the position of the thin film reflection are visible. Thus, only the surface region is affected by the thermal treatment while the crystallographic properties remain unchanged.

Throughout our *in-situ* spectroscopy and *ex-situ* topological investigations addressing the blocking space charge effect, the thin film samples were processed at low temperatures of T = 400°C and oxygen partial pressures of $p(O_2)$ = 0.1 mbar. In order to rule out the significant segregation of host cations under oxidizing conditions that may influence the exsolution response by the formation of a secondary phase at the perovskite surface, we show the ratio of the integrated peak areas of the Ti 2p and Sr 3d core-level spectra recorded at different points of time during the oxidizing annealing step of protocol 2 of our NAP-XPS investigations (*cf.* Fig. 2a,b) in Fig. S4a. As can be seen, no considerable changes of the total peak area ratio are detected over the entire annealing time indicating that no dense cover layer was formed *i.e.* that no segregation of either cation is induced by the oxidizing treatment. For comparison, we calculate the theoretical attenuation of the Ti 2p XPS core-level intensity assuming the presence of a dense SrO surface layer of different thickness. For this purpose the inelastic mean free path was calculated by the QUASES-IMFP software using the TPP2M formula.[3] On the basis of the IMFP, the Ti 2p core-level intensity can be estimated to be attenuated to 63% of the intensity acquired from a pristine STNNi surface if an SrO blanketing layer of only 1 nm thickness was present. If an SrO surface layer of 2 nm thickness was present at the STNNi surface an attenuation of the Ti 2p intensity to 39% of the initial intensity would be expected.

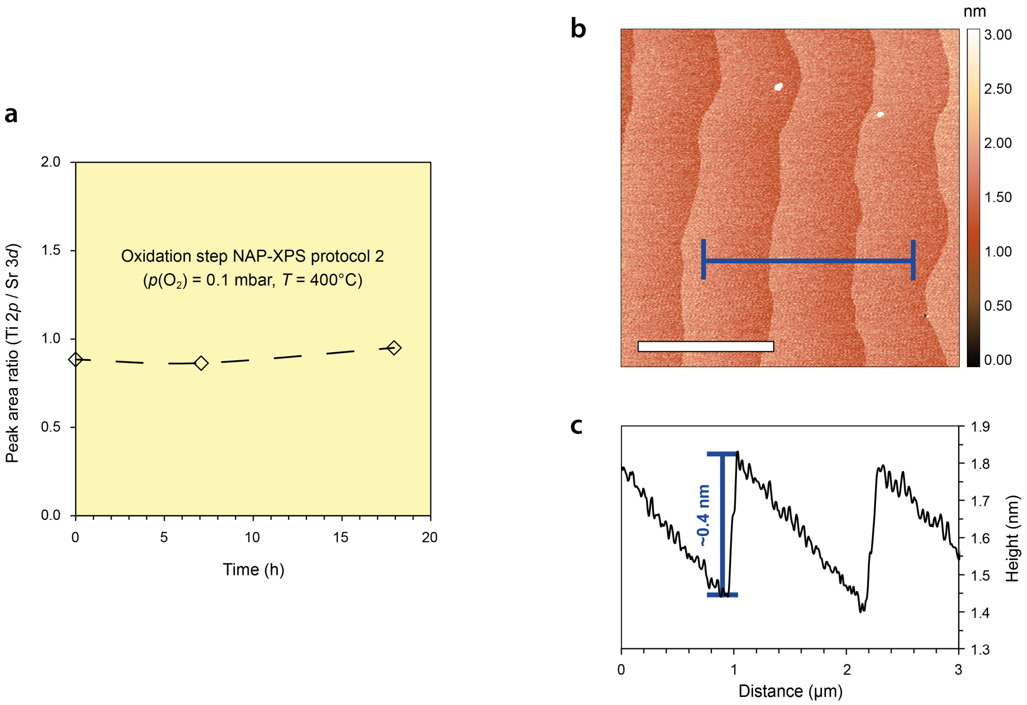

Fig. S4| (a) Ratio of the integrated Ti 2*p* and Sr 3*d* peak areas recorded at different points of time during oxidizing treatment of STNNi by near ambient pressure X-ray photoelectron spectroscopy. A Shirley-type background was subtracted. (b) Atomic force microscopy of the STNNi surface after oxidizing annealing at T = 400°C and $p(O_2)$= 0.108 mbar for 60 min. After oxidizing annealing at T = 400°C and $p(O_2)$= 0.108 mbar for 60 min the STNNi surface exhibits a smooth surface morphology with a distinct step terrace structure. The scale bar denotes 2 µm. (c) A line profile extracted from (b) reveals a terrace step height of ~0.4 nm equal to the perovskite unit cell and well comparable to the as-prepared state of the sample.

Furthermore, Fig. S4 shows a representative AFM scan (Fig. S4b) and the according line profile across the terrace step structure of an STNNi thin film after oxidizing annealing (Fig. S4c). As can be seen, the terrace step structure is preserved after the oxidation and the step height is ~0.4 nm equal to the height of the as-prepared perovskite unit cell. Therefore, no indications for extended cation segregation under oxidizing conditions can be detected. As we show in the main manuscript the time span of 60 min oxidizing treatment already results in a significant suppression of the exsolution response (Fig. 2d), indicating that not a stoichiometric effect but a space charge effect is present.

Supplementary Note 4: Epitaxial control of the surface termination

In Fig. S5 we show the synthesis of an STNNi thin film sample with an engineered SrO-termination layer (one atomic layer in thickness) and the morphology evolution during metal exsolution. After deposition of a 20 nm thick STNNi thin film with monolayer precision via RHEED-PLD (Fig. S5a), an SrO-termination layer is fabricated by *in-situ* ablation of a ceramic SrO_2 target (Fig. S5b), *i.e.* without exposure to air when changing between the target materials. In this way, the surface termination can be controlled, synthesizing a SrO-terminated sample to investigate its' influence on the exsolution response. Notably, the native surface of as-deposited STNNi is likely to exhibit a mixed SrO/TiO_2 termination. The termination change is apparent on the basis of the intensity evolution of the RHEED surface electron diffraction pattern (Fig. S5c), were the change in the relative intensity of the (0 0) specular and the (1 0) / (-1 0) diffraction spots provides information about changes of the chemical composition of the atomic termination layer.[4–7] Here, the intensity increase of the diffraction spots relative to the intensity of the central specular spot indicates the formation of an SrO surface termination layer (Fig. S5c, d). Therefore, RHEED-PLD enables the precise deposition of one monolayer of SrO by *in-situ* monitoring of the relative intensity change, which is shown in Fig. 5d.

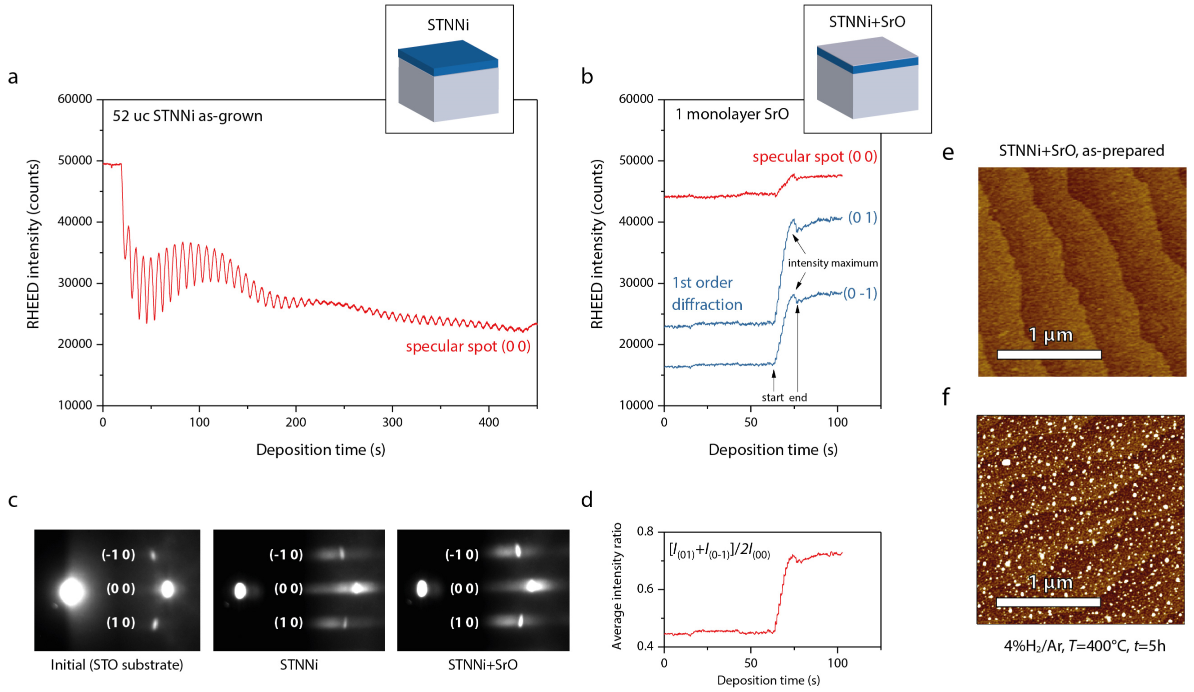

Fig. S5| Synthesis of epitaxial STNNi thin films with an engineered SrO surface termination layer. (a) *In-situ* RHEED monitoring of the PLD of 52 monolayers (20 nm) of STNNi in a layer-by-layer deposition mode. (b) *In-situ* RHEED monitoring of the PLD of one monolayer of SrO. (c) RHEED surface electron diffraction pattern obtained from the TiO_2-terminated STO substrate (left), the as-deposited STNNi surface (center) and after deposition of one monolayer of SrO (right). (d) The average intensity ratio between the first order diffraction spots and the specular spot during SrO deposition revealing a change in their relative intensity. (e) Atomic force microscopy imaging of the as-deposited SrO-terminated STNNi surface and (f) of the same surface after reducing annealing. The reducing annealing was performed in a continuous flow of a 4% H_2/Ar gas mixture at $T = 400°C$ for $t = 5$ h.

As can be seen in Fig. S5e, the growth of STNNi with an engineered SrO termination layer can be achieved with smooth surface morphology of the thin films, yielding ideal model samples for studying the impact of SrO surface termination on the exsolution behavior. Importantly, the SrO-terminated STNNi thin film

fabricated by sequential deposition of STNNi and SrO represents a surface with altered termination layer, while the formation of negatively charged strontium vacancies during the fabrication is negligible compared to samples that have experienced thermal oxidation. In contrast, oxidizing pre-annealing results in formation of a strong electrostatic field at the surface as well as space charge driven enrichment of trace amounts of Sr species, as discussed in our main manuscript. After reducing annealing of the sample at equal conditions to the oxygen pre-annealing study presented in our manuscript (5 h, 400°C, 4% H_2/Ar), the surface morphology is investigated by atomic force microscopy (Fig. S2f). Our findings show that a SrO-termination layer, fabricated by PLD, cannot suppress the exsolution response at the STNNi surface. While a high density of exsolved nanoparticles is visible at the SrO-terminated STNNi surface, the introduction of a negative surface potential results in a strong suppression of nanoparticle exsolution considering the same time-temperature window (*cf.* Fig. 2d and Figure S3a). This indicates that not a change in the termination chemistry results in a suppression of the exsolution process, but the space charge region that is introduced upon oxidizing annealing. Yet, a clear influence of SrO-termination on the exsolution and nucleation dynamics on the surface is apparent (as expected), where a larger nanoparticle density of smaller average size, but with comparable sum of the nanoparticle volume around 1E-22 m3·µm-2 is detected as for sample that have not experienced oxidizing pre-annealing, which resulted in a drop of exsolution volume by one order of magnitude.

Supplementary Note 5: Redox response of STNNi

In Fig. S6a an extended measurement protocol applied during NAP-XPS investigations is displayed including multiple consecutive reducing and oxidizing steps (the first part is equal to protocol 1 as discussed in the main manuscript, Fig. 2a). After introduction of hydrogen to the NAP-XPS chamber, formation of metallic Ni species is apparent based on the emergence of a low-binding energy signal. By switching of the ambient gas from hydrogen to oxygen, the nanoparticles are oxidized and the metallic signal is absent. After a second reduction step, again a significant metal signal is detected.

Here, the main oxide peak is centered at B.E. ~855.6 eV, while the main peak of the metallic Ni $2p_{3/2}$ state is detected at a lower relative binding energy centered at B.E. ~851.9 eV.[8–10] The absolute binding energy of the metal signal may be influenced by the size of the supported nanoparticles and by interactions with the oxide support.[11–14] Notably, oxidation typically results in an increase of the nanoparticle volume, which may result in an overall increase of the Ni signal intensity in comparison to the initial formation of the nanoparticles.

Depending on the ambient atmosphere, significant shifts in the binding energy relative to UHV conditions (position denoted by dashed lines) can be observed based on the Ti 2*p*, Sr 3*d*, O 1*s* and Nb 3*d* core-level spectra (Fig. S6b). Please note that the redox equilibria are not activated at room temperature *i.e.*, UHV conditions and hence the binding energy position at UHV conditions is used only as a relative reference value. Since the spectra are obtained from the perovskite support, the evident shift in binding energy is not related to the nanoparticle size as observed for the metallic Ni 2*p* signal. The apparent peak shifts also cannot be understood in terms of sample charging due to insufficient supply of electrons from the thin film bulk. Such limited charge compensation would result in a continuous shift toward larger binding energies and for *n*-STO would be expected to be most pronounced in oxidizing conditions, whereas the observed shift upon oxidation is in the opposite direction. The shift in binding energy hence is directly related to the formation of space charge regions at the perovskite surface.

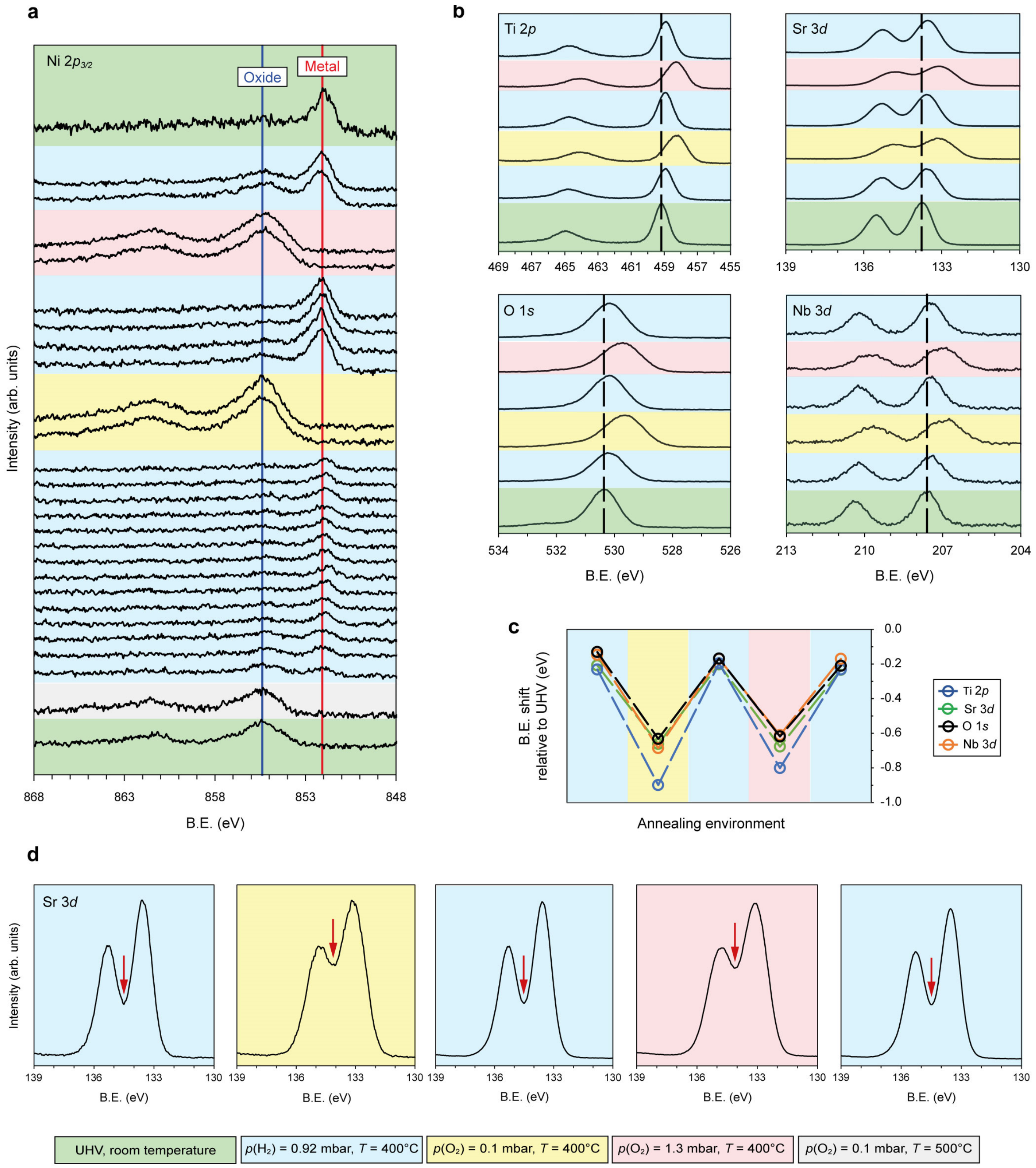

Fig. S6| Near ambient pressure x-ray photoelectron spectroscopy. (a) Extended NAP-XPS measurement protocol showing the Ni 2*p*3/2 core-level spectrum under different redox conditions. The binding energy was corrected to the Ti 2*p* position. (b) Representative XPS core-level spectra of the Ti 2*p*, Sr 3*d*, O 1*s*, and Nb 3*d* regions recorded under UHV conditions and under ambient gas atmosphere. No correction of the binding energy position is applied for (b). The dashed line denotes UHV position. Sample conditions are denoted by the color code given below. (c) Based on shifts in the binding energy (determined from (b)) relative to ultra-high-vacuum conditions, the formation of a surface space charge region can be observed. The space charge potential is different for oxidizing and reducing conditions as it depends on the respective electric and ionic reconstruction in the surface region and reproducibly forms under repeated redox cycling. (d) Detailed comparison of representative Sr 3*d* core-level spectra recorded under repeated redox cycling, where a reversible change in the width of the photoemission signals is visible for different annealing environments that accompanies the binding energy shift depicted in (b,c). Red arrows highlight changes in the intensity valley between the Sr 3*d*3/2 and Sr 3*d*5/2 doublet.

The broadening of the NAP-XPS spectra may have different origins, some of which are directly related to the formation of SCRs at the surface. Here, peak broadening may be induced by the potential gradient at the surface[15–19] secondary phase formation as well as scattering effects with the ambient oxygen or hydrogen molecules. The rigid peak broadening can be rationalized by the fact that the surface potential drops across the width of the surface region and results in slight shifts in the binding energy that changes with depth. Therefore, the core-level XPS spectra consist of a large number of spectra with slightly different binding energies that manifest in a broadening of the recorded spectra.[18,19]

Remarkably, the apparent peak broadening is most pronounced for the Sr 3*d* region under oxidizing conditions, which results in a significantly flattened intensity valley in between the Sr 3*d* (Sr $3d_{5/2}$ and Sr $3d_{3/2}$) doublet. This observation may indicate the evolution of a minor SrO or $Sr(OH)_2$ surface phase, which is contributing to the signal in form of a second, superimposed doublet of slightly different binding energy. The formation of a Sr-rich surface phase under oxidizing conditions is a direct consequence of the defect chemistry of the perovskite host lattice and strongly entangled with the formation of surface space charge regions as well as the observed passivation effect. Notably, repeated cycling results in reversible and reproducible binding energy shifts accompanied by respective changes in the width of the photoemission signals (*cf.* Supplementary Fig. S6).[15,20,17] Extensive broadening of the Sr 3*d* core-level spectrum under oxidizing conditions may indicate the formation of a Sr-rich (SrO or $Sr(OH)_2$) surface phase in agreement with the formation of a negative surface potential by Sr vacancies kinetically trapped in the near-surface region.[21]

The detected binding energy shifts and broadening of the photoemission signals are reproducible and fully reversible, where several cycles of redox annealing have been conducted (Fig. S6b,c), indicating that the space charge formation is driven by the thermodynamic equilibration at the different oxygen environments. A detailed comparison of the Sr 3*d* core-level spectra under different annealing environments is given in Fig. S6d. Here, red arrows highlight changes in the intensity valley between the Sr $3d_{5/2}$ and Sr $3d_{3/2}$ signals that compose the Sr 3*d* core-level signature and that are directly correlated to changes in the width of photoelectron emission signal.

Supplementary Note 6: Surface engineering of the surface redox chemistry

Epitaxial growth allows to precisely tailor the surface chemistry of the exsolution-active STNNi thin film by deposition of a top layer with a monolayer thickness in a well-defined manner (Fig. S7). After reducing thermal treatment, the formation of Ni nanoparticles can be observed at the surface of the top layer. The Ni dopants hence are transported through the originally Ni-free top layer material.

Reducing treatment of the stack samples after thermal activation of the redox equilibria by oxidizing pre-annealing significantly modifies the exsolution response of Ni-dopants, depending on the redox chemistry of the top layer. A top layer with donor-type doping results in surface passivation *i.e.* a retarded exsolution response (in agreement with the behavior of the STNNi film itself). In contrast, a top layer with acceptor-type doping does not result in surface passivation (*cf.* Fig. 3d, main manuscript). Please note that the evaluation of the exsolution response is based on the averaged total volume of the exsolved nanoparticles. Note that the total nanoparticle volume is determined by the particle size and the nanoparticle density.

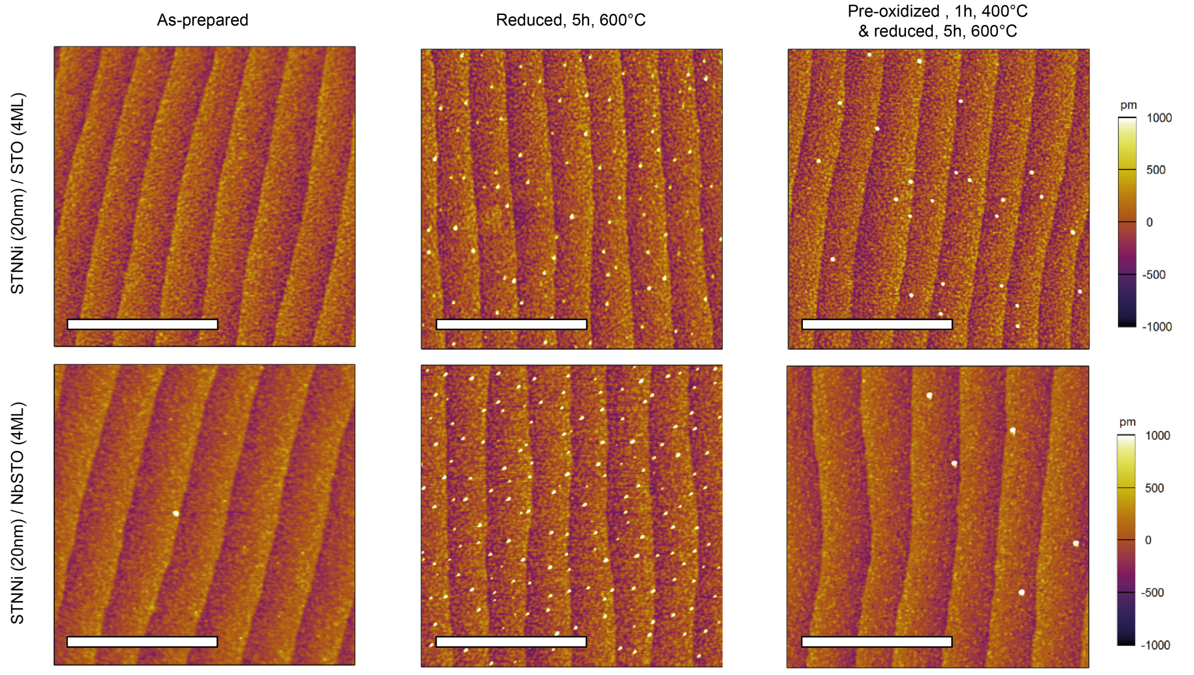

Fig. S7| Representative AFM scans showing the surface morphology of stack samples, combining an STNNi bottom layer (20nm) and a top layer of four monolayers. Nb-doped $SrTiO_3$ (donor-type) and undoped $SrTiO_3$ (acceptor-type) is deposited as top layer material. Surface morphology is shown for samples in the as-prepared state and after reducing thermal treatment (reducing conditions: 4% H_2/Ar, $T = 400$°C, $t = 5$ h), comparing samples without oxidizing pre-annealing and after pre-annealing (oxidizing conditions: $p(O_2)$= 0.108 mbar, $T = 400$°C). The exsolution response is determined by the redox chemistry of the top layer material, *i.e.* a passivation effect is detected for the donor-doped (pre-oxidized) surface. The AFM scan size is 2x2 μm^2 and scale bars 1 μm respectively.

Supplementary Note 7: Finite-element electrostatic space charge simulations

Starting point for the space charge simulations is the bulk defect chemistry model of $SrTiO_3$ yielding the concentrations of electrons, electron holes and oxygen vacancies in the bulk as a function of temperature, ambient oxygen partial pressure (oxygen activity, aO_2) and dopant concentration[20].

For this, we use the intrinsic generation of electron-hole pairs via band gap excitation

$$e + h \rightleftarrows \mathrm{nil} \tag{1}$$

and the corresponding law of mass action

$$np = K_{\mathrm{i}}^{0} \exp\left(-\frac{\Delta E_{\mathrm{g}}}{k_{\mathrm{B}}T}\right). \tag{2}$$

Here, n (p) denote electron (hole) concentration, K_{i}^{0} the reaction constant prefactor for the intrinsic electron-hole pair equilibrium, and ΔE_{g} the band gap of $SrTiO_3$. We moreover consider the oxygen exchange equilibrium

$$\mathrm{O}_{\mathrm{O}}^{\mathrm{x}} \rightleftarrows \mathrm{V}_{\mathrm{O}}^{\cdot\cdot} + 2\mathrm{e} + \frac{1}{2}\mathrm{O}_2. \tag{3}$$

In thermodynamic equilibrium, this renders the law of mass action

$$n^2 c_{\mathrm{V}_{\mathrm{O}}^{\cdot\cdot}} (a\mathrm{O}_2)^{-1/2} = K_0^{\mathrm{red}} \exp\left(-\frac{\Delta H_{\mathrm{red}}}{k_{\mathrm{B}}T}\right). \tag{4}$$

Here, ΔH_{red} denotes the enthalpy of reduction of the material, *i.e.* the actual enthalpy required to form an oxygen vacancy, and $c_{\mathrm{V}_{\mathrm{O}}^{\cdot\cdot}}$ the concentration of doubly ionized oxygen vacancies. Eq. (4) implies the oxygen pressure/activity dependence of the oxygen vacancy concentration and the electron concentration in oxides. In the bulk, the three variable parameters n, p and $c_{\mathrm{V}_{\mathrm{O}}^{\cdot\cdot}}$ are coupled *via* the charge-neutrality condition

$$2c_{\mathrm{V}_{\mathrm{O}}^{\cdot\cdot}} + c_{\mathrm{Nb}_{\mathrm{eff}}^{\cdot}} + p - n = 0, \tag{5}$$

allowing to solve for all defect concentrations at a given T and aO_2. Note that the reaction constants and enthalpies are available in the literature[22,20,23], as listed in table S1.

At moderate temperature, space charge formation at the surface of $SrTiO_3$ is described by 1) formation of surface strontium vacancies *via* the partial Schottky equilibrium[21,20,15] and 2) by a reduction of the local enthalpy of formation for oxygen vacancies[24–26], which is reduced by $\Delta g = -1.4$ eV as compared to the bulk value. For the surface-terminating unit cell of the STNNi layer we therefore additionally assume an active partial Schottky equilibrium

$$\mathrm{Sr}_{\mathrm{Sr}}^{\mathrm{x}} + \mathrm{O}_{\mathrm{O}}^{\mathrm{x}} \rightleftarrows \mathrm{V}_{\mathrm{O}}^{\cdot\cdot} + \mathrm{V}_{\mathrm{Sr}}^{\prime\prime} + (\mathrm{SrO})_{\mathrm{sp.}} \tag{6}$$

and the corresponding law of mass action

$$c_{\mathrm{V}_{\mathrm{Sr}}^{\prime\prime}} c_{\mathrm{V}_{\mathrm{O}}^{\cdot\cdot}} = K_0^{\mathrm{S}} \exp\left(-\frac{\Delta H_{\mathrm{S}}}{k_{\mathrm{B}}T}\right). \tag{7}$$

The active surface Schottky-equilibrium results in the formation of surface strontium vacancies in oxidizing conditions which yield an electron depletion layer close the surface and a repelling electric field for acceptor-type dopants, such as Ni. For the oxygen exchange equilibrium at the surface, we moreover use $\Delta H_{\mathrm{red}}(\mathrm{surface}) = \Delta H_{\mathrm{red}}(\mathrm{bulk}) - 1.4$ eV. This condition reflects an energetically favoured formation of oxygen vacancies in the surface-near region, well discussed in the literature and essentially allows the inversion of the space charge potential at low oxygen activity, where oxygen vacancies become the dominant defect species in highly donor doped $SrTiO_3$.

The dedicated formulation of surface equilibria results in differing defect concentrations in bulk and surface at a given temperature and oxygen activity. As a result of this, a redistribution of defects via diffusion and drift is triggered which essentially leads to a balanced space charge equilibrium, in which diffusive currents driven *via* concentration gradient and drift currents driven *via* local electric fields vanish. This equilibrium moreover corresponds to the minimum in Gibbs free energy of the system, defining the thermodynamic equilibrium state of the space charge layer. In order to solve the space charge potential $\phi(x)$ we self-consistently solve eqs. (2), (4) and (7) together the boundary conditions of

$$c_{\mathrm{def}}(x) = c_{\mathrm{def}}^{\mathrm{bulk}} \exp\left(\frac{e\varphi(x)}{k_{\mathrm{B}}T}\right) \tag{8}$$

$$\varepsilon\varepsilon_{\mathrm{r}} \frac{d\varphi(x=0)}{dx} = \frac{Q}{\mathrm{A}} \tag{9}$$

$$\varepsilon\varepsilon_{\mathrm{r}} \frac{d\varphi(x=\infty)}{dx} = 0 \tag{10}$$

$$\varepsilon_0\varepsilon_{\mathrm{r}} \frac{d^2\varphi(x)}{dx^2} = e\left(2c_{\mathrm{V_O^{\cdot\cdot}}}(x) + c_{\mathrm{Nb_{eff}^{\cdot}}} + p(x) - n(x)\right) \tag{11}$$

Here, equations (9), (10) reflect Gauss law evaluated at the surface ($x = 0$) and far from the surface, where the electric field vanishes (global charge neutrality). At the surface, the electric field $\frac{d\varphi(x=0)}{\mathrm{dx}}$ is given by the surface charge

$$\frac{Q}{\mathrm{A}}(\mathrm{surface}) = ec\left(2c_{\mathrm{V_O^{\cdot\cdot}}}(0) + c_{\mathrm{Nb_{eff}^{\cdot}}} + p(0) - n(0) - 2c_{\mathrm{V_{Sr}''}}(0)\right). \tag{12}$$

Here, c denotes the lattice constant of $SrTiO_3$ and the surface concentrations $c_{\mathrm{V_O^{\cdot\cdot}}}(0)$, $c_{\mathrm{V_{Sr}''}}(0)$ obey the surface equilibria defined in equation (7) and (4), considering a reduced reduction enthalpy. Equation (11) reflects the Poisson equation, whereas the local charge density is given by the sum of local defect concentrations within the space charge layer.

All equation can be solved numerically using a finite-element approach, revealing the established surface space charge potential $\phi(x)$ as shown in the main paper, as well as all defect concentration profiles within the surface layer. Table S1 summarizes all numerical values used for space charge calculations as available in the listed literature.

Table S1 | Thermodynamic data for $SrTiO_3$.

		Thermodynamic data	Reference
**Bulk**	K_i^0 / cm^{-6}	$1.43 \times 10^{33} \times (T/\mathrm{K})^3$	22
	ΔE_g / eV	$3.17 - 5.66 \times 10^{-4} \times (T/\mathrm{K})$	22
	K_{red}^0 / cm^{-9}bar$^{-1/2}$	5×10^{71}	22
	ΔH_{red} / eV	5.7	27
**Surface**	K_i^0 / cm^{-6}	$1.43 \times 10^{33} \times (T/\mathrm{K})^3$	22
	ΔE_g / eV	$3.17 - 5.66 \times 10^{-4} \times (T/\mathrm{K})$	22
	K_{red}^0 / cm^{-9}bar$^{-1/2}$	5×10^{71}	22
	ΔH_{red} / eV	4.3	25,26
	K_S^0 / cm^{-6}	3×10^{44}	22,20
	ΔH_S / eV	2.5	22,20
**SCL**	$\varepsilon(T)$	$\frac{78400}{(T/\mathrm{K}) - 28}$	20

References

1. Weber, M. L. *et al.* Exsolution of Embedded Nanoparticles in Defect Engineered Perovskite Layers. *ACS nano* **15,** 4546–4560 (2021).
2. Weber, M. L. *et al.* Reversibility limitations of metal exsolution reactions in niobium and nickel co-doped strontium titanate. *J. Mater. Chem. A* (2023).
3. Tanuma, S., Powell, C. J. & Penn, D. R. Calculations of electron inelastic mean free paths. V. Data for 14 organic compounds over the 50-2000 eV range. *Surf. Interface Anal.* **21,** 165–176 (1994).
4. Sun, H. Y. *et al.* Chemically specific termination control of oxide interfaces via layer-by-layer mean inner potential engineering. *Nature communications* **9,** 2965 (2018).
5. Lei, Q. *et al.* Constructing oxide interfaces and heterostructures by atomic layer-by-layer laser molecular beam epitaxy. *npj Quant Mater* **2** (2017).
6. Yan, H. *et al.* Stoichiometry and Termination Control of $LaAlO_3/SrTiO_3$ Bilayer Interfaces. *Adv. Mater. Interfaces* **8,** 2001477 (2021).
7. Baeumer, C. *et al.* Surface Termination Conversion during SrTiO3 Thin Film Growth Revealed by X-ray Photoelectron Spectroscopy. *Scientific reports* **5,** 11829 (2015).
8. Biesinger, M. C. *et al.* Resolving surface chemical states in XPS analysis of first row transition metals, oxides and hydroxides: Cr, Mn, Fe, Co and Ni. *Applied Surface Science* **257,** 2717–2730 (2011).
9. Payne, B. P., Biesinger, M. C. & McIntyre, N. S. Use of oxygen/nickel ratios in the XPS characterisation of oxide phases on nickel metal and nickel alloy surfaces. *Journal of Electron Spectroscopy and Related Phenomena* **185,** 159–166 (2012).
10. Biesinger, M. C., Payne, B. P., Lau, L. W. M., Gerson, A. & St. Smart, R. C. X-ray photoelectron spectroscopic chemical state quantification of mixed nickel metal, oxide and hydroxide systems. *Surf. Interface Anal.* **41,** 324–332 (2009).
11. Mason, M. G. Electronic structure of supported small metal clusters. *Phys. Rev. B* **27,** 748–762 (1983).
12. Wertheim, G. K. Core-electron binding energies in free and supported metal clusters. *Z. Physik B - Condensed Matter* **66,** 53–63 (1987).
13. Wertheim, G. K. Electronic structure of metal clusters. *Z Phys D - Atoms, Molecules and Clusters* **12,** 319–326 (1989).
14. Richter, B., Kuhlenbeck, H., Freund, H. J. & Bagus, P. S. Cluster core-level binding-energy shifts: the role of lattice strain. *Phys. Rev. Lett.* **93,** 26805 (2004).
15. Andrä, M. *et al.* Chemical control of the electrical surface properties in donor-doped transition metal oxides. *Phys. Rev. Materials* **3** (2019).
16. Chambers, S. A., Du, Y., Comes, R. B., Spurgeon, S. R. & Sushko, P. V. The effects of core-level broadening in determining band alignment at the epitaxial $SrTiO_3$ (001)/p-Ge(001) heterojunction. *Appl. Phys. Lett.* **110,** 82104 (2017).
17. Rose, M.-A. *et al.* Identifying Ionic and Electronic Charge Transfer at Oxide Heterointerfaces. *Advanced materials (Deerfield Beach, Fla.),* e2004132 (2020).
18. Chambers, S. A. *et al.* Instability, intermixing and electronic structure at the epitaxial $LaAlO_3/SrTiO_3$(001) heterojunction. *Surface Science Reports* **65,** 317–352 (2010).
19. Sushko, P. V. & Chambers, S. A. Extracting band edge profiles at semiconductor heterostructures from hard-x-ray core-level photoelectron spectra. *Scientific reports* **10,** 13028 (2020).

20. Meyer, R., Zurhelle, A. F., Souza, R. A. de, Waser, R. & Gunkel, F. Dynamics of the metal-insulator transition of donor-doped $SrTiO_3$. *Phys. Rev. B* **94** (2016).

21. Andrä, M. *et al.* Oxygen partial pressure dependence of surface space charge formation in donor-doped $SrTiO_3$. *APL Materials* **5,** 56106 (2017).

22. Moos, R. & Hardtl, K. H. Defect Chemistry of Donor-Doped and Undoped Strontium Titanate Ceramics between 1000° and 1400°C. *J American Ceramic Society* **80,** 2549–2562 (1997).

23. Zurhelle, A. F., Christensen, D. V., Menzel, S. & Gunkel, F. Dynamics of the spatial separation of electrons and mobile oxygen vacancies in oxide heterostructures. *Phys. Rev. Materials* **4** (2020).

24. Metlenko, V. *et al.* Do dislocations act as atomic autobahns for oxygen in the perovskite oxide $SrTiO_3$? *Nanoscale* **6,** 12864–12876 (2014).

25. Souza, R. A. de. The formation of equilibrium space-charge zones at grain boundaries in the perovskite oxide $SrTiO_3$. *Physical chemistry chemical physics : PCCP* **11,** 9939–9969 (2009).

26. Souza, R. A. de, Gunkel, F., Hoffmann-Eifert, S. & Dittmann, R. Finite-size versus interface-proximity effects in thin-film epitaxial $SrTiO_3$. *Phys. Rev. B* **89** (2014).

27. Ohly, C., Hoffmann-Eifert, S., Guo, X., Schubert, J. & Waser, R. Electrical Conductivity of Epitaxial SrTiO3 Thin Films as a Function of Oxygen Partial Pressure and Temperature. *J American Ceramic Society* **89,** 2845–2852 (2006).
